# Remdesivir for patients with Coronavirus disease 2019 pneumonia requiring high oxygen support

**DOI:** 10.5339/qmj.2022.25

**Published:** 2022-06-16

**Authors:** Rim S. Alibrahim, Eman Z. Elmekaty, Mohamed Z. I. Elmekaty, Mohammad Edbais, Mohammed Alkhatib, Joanne Daghfal, Muna A. Almaslamani, Ali S. Omrani

**Affiliations:** ^1^Department of Pharmacy, Communicable Diseases Center, Hamad Medical Corporation, Doha, Qatar E-mail: aomrani@hamad.qa; ^2^Department of Pediatrics, Medical Education, Hamad Medical Corporation, Doha, Qatar; ^3^Department of Pharmacy, Rumailah Hospital, Hamad Medical Corporation, Doha Qatar; ^4^Department of Medicine, Hamad General Hospital, Hamad Medical Corporation, Doha Qatar; ^5^Communicable Diseases Center, Hamad Medical Corporation, Doha, Qatar; ^6^Division of Infectious Diseases, Department of Medicine, Hamad Medical Corporation, Doha, Qatar#These authors contributed equally to this work

**Keywords:** remdesivir, coronavirus, severe pneumonia, intensive care unit, mechanical ventilation, bradycardia

## Abstract

Background: Treatment options for patients with critical Coronavirus Disease 2019 (COVID-19) are limited. This study aimed to describe the clinical characteristics and outcomes associated with remdesivir therapy in patients with COVID-19 who require non-invasive (NIV) ventilation or invasive mechanical ventilation (IMV).

Methods: Data were retrospectively extracted for adults with COVID-19 confirmed using polymerase chain reaction (PCR) between August 1, 2020 and January 28, 2021 who received ≥ 48 hours of remdesivir therapy while on NIV or IMV. Clinical improvement was defined as two-category improvement on an eight-point ordinal severity scale.

Results: A total of 133 individuals were included, of which 114 (85.7%) were on NIV and 19 (14.3%) were on IMV at the time of remdesivir initiation. The majority of the patients were males (62.4%), and the median age was 56 years. All the patients received concomitant dexamethasone therapy. Remdesivir treatment was commenced after a median of 7 days from onset of symptoms and was continued for a median of 5 days.

Clinical improvement within 28 days was achieved in 101 patients (75.9%); among which, 78.1% and 63.2% were subjected to baseline NIV and IMV, respectively. Among the 11 (8.3%) patients who died of any cause by day 28, 9 (7.9%) and 2 (10.5%) were subjected to baseline NIV and IMV, respectively. The most frequent adverse events were sinus bradycardia (21, 13.1%) and alanine transaminase increase (18, 11.3%). Almost all adverse events were classified as Grades 1–3.

Conclusion: The use of remdesivir in combination with systemic corticosteroids is associated with high recovery rates and low all-cause mortality in patients with COVID-19 pneumonia who require NIV or IMV. The results need confirmation from clinical trials of appropriate design and size.

## Introduction

Approximately 10%–20% of patients with Coronavirus Disease 2019 (COVID-19), which is caused by Severe Acute Respiratory Syndrome Coronavirus-2 (SARS-CoV-2), develop a severe illness requiring high levels of organ support.^1^ Immune modulating therapies, such as systemic corticosteroids and tocilizumab, are associated with improved clinical outcomes in the patients with critical illness.^2,3^ However, evidence on the role for antiviral therapy in such patients is limited.

Remdesivir, a nucleotide analog inhibitor of SARS-CoV-2 RNA-dependent RNA polymerase, was evaluated in randomized clinical trials as a potential therapeutic agent for patients with COVID-19.^4^ Adaptive COVID-19 Treatment Trial (ACTT)-1 is a double-blind, randomized, placebo-controlled, clinical trial of 1,062 patients with COVID-19 who were randomized to receiving remdesivir or placebo in addition to standard care. The overall median recovery time for the patients who received remdesivir was 10 days, which is shorter than the 15 days for those who received placebo [rate ratio (RR) for recovery, 1.29; 95% confidence interval (CI) 1.12–1.49; P <  0.001]. However, remdesivir was not associated with a higher probability of recovery in the subsets of patients who required non-invasive ventilation (NIV) (RR 1.09, 95% CI 0.76–1.57) or invasive mechanical ventilation (IMV) (RR 0.98, 95% CI 0.70–1.36) at the time of enrollment.^5^ Moreover, remdesivir was not associated with reduced all-cause mortality at 28 days in ACTT-1 and the World Health Organization's SOLIDARITY trial.^4^ Current COVID-19 management guidelines contain conflicting recommendations on the use of remdesivir for the treatment of patients with COVID-19 who require NIV or IMV.^6-8^

Remdesivir was introduced as a treatment option for patients with COVID-19 in Qatar in August 2020. This study aimed to describe the clinical characteristics and outcomes associated with remdesivir therapy in patients with laboratory-confirmed COVID-19 who require NIV or IMV.

## Methods

This retrospective cohort study was undertaken at Hamad Medical Corporation, the provider of all COVID-19 medical care for the entire population of Qatar. Patients aged ≥ 18 years with polymerase chain reaction (PCR)-confirmed COVID-19 and radiologically verified pneumonia who visited between August 1, 2020 and January 28, 2021 were included in this study if they had received ≥ 48 hours of remdesivir therapy and were on NIV, including high-flow nasal oxygen (HFNO), or IMV at the time of remdesivir initiation. Remdesivir was administered intravenously at 200 mg once daily on the first day, followed by 100 mg daily for an additional 4–9 days.

Data were retrospectively extracted from the electronic health records by one set of investigators and subsequently independently validated by another set of investigators. COVID-19 severity was categorized according to an eight-point ordinal scale as follows: 1, not hospitalized and without limitations of activities; 2, not hospitalized but has limitation of activities and requires oxygen, or both; 3, hospitalized, not requiring supplemental oxygen, and no longer requiring ongoing medical care; 4, hospitalized, not requiring supplemental oxygen but requiring ongoing medical care; 5, hospitalized and requiring supplemental oxygen; 6, hospitalized and requiring NIV or HFNO; 7, hospitalized and receiving IMV or extracorporeal membrane oxygenation; and 8, death.^5^ The primary outcome was the proportion of patients with clinical improvement, defined as two-category improvement on the ordinal severity scale, within 28 days. Adverse events were defined and graded according to Common Terminology Criteria for Adverse Events Version 5.^9^

Categorical data were summarized as numbers (percentages), and continuous data were presented as medians and interquartile ranges (IQR). All data were anonymized before analysis. This study was approved by the Institutional Review Board at Hamad Medical Corporation (MRC-01-21-479).

## Results

The eligibility criteria were met by 133 individuals, of which 114 (85.7%) were on NIV and 19 (14.3%) were on IMV at the time of remdesivir initiation. The majority of patients were males (83, 62.4%), and the median age was 56 years (IQR, 44–64). The most frequent co-existing medical conditions were diabetes mellitus (58, 43.6%), hypertension (63, 47.4%), and chronic heart disease (20, 15%), and the median body mass index was 28.9?kg/m^2^ (IQR, 25.7–33.1). Cough (111, 83.5%), fever (106, 79.7%), and dyspnea (98, 73.7%) were the most common presenting symptoms. Pneumonia was bilateral in the majority of the patients (129, 96.7%).

All the patients received concomitant intravenous dexamethasone therapy (6–8 mg daily for up to 10 days), and 33 (24.8%) individuals were also prescribed with tocilizumab. Remdesivir treatment commenced after a median of 7 days (IQR 5–10) from onset of symptoms or 1 day (IQR 1–3) from hospitalization and was continued for a median of 5 days (IQR 5–6). Median baseline findings included heart rate at 96 per minute (IQR 86–106), respiratory rate at 34 per minute (IQR 30–40), oxygen saturation at 90% (IQR 88%–93%), peripheral lymphocyte count at 0.7x10^9^/L (IQR 0.5–1.0), C-reactive protein concentration at 91.5 mg/L (IQR 46.5–148.5), and serum ferritin level at 0.71 μg/L IQR (0.46–1.30).

**Figure 1.**
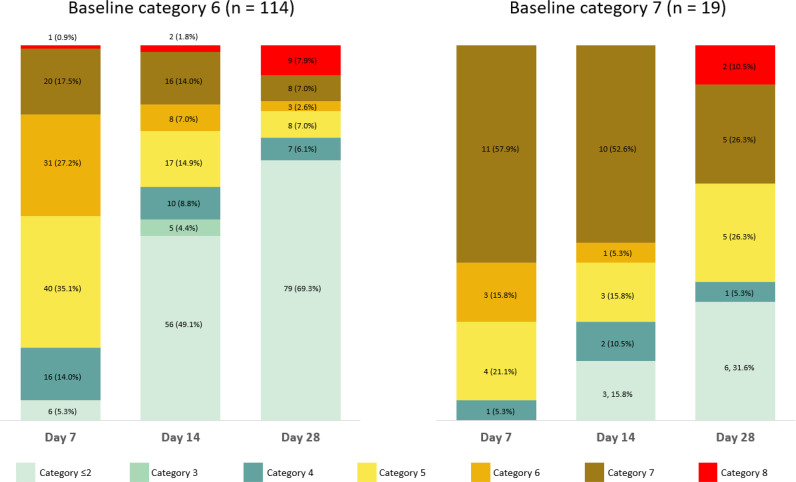
Coronavirus Disease 2019 severity score by baseline oxygen support status. Category 1, not hospitalized and without limitations of activities; Category 2, not hospitalized but has limitation of activities, requiring oxygen, or both; Category 3, hospitalized, not requiring supplemental oxygen and no longer requiring ongoing medical; Category 4, hospitalized, not requiring supplemental oxygen but requiring ongoing medical care; Category 5, hospitalized and requiring supplemental oxygen; Category 6, hospitalized and requiring non-invasive ventilation or use of high-flow nasal oxygen devices; Category 7, hospitalized and receiving invasive mechanical ventilation or extracorporeal membrane oxygenation; Category 8, death

Clinical improvement within 28 days was achieved in 101 patients (75.9%); among which, 89 (78.1%) were on baseline NIV and 12 (63.2%) were on baseline IMV. Among the 11 (8.3%) patients who died of any cause by day 28, 9 (7.9%) and 2 (10.5%) were on baseline category 6 and category 7 disease severity score, respectively. The ordinal severity categorizations of the study population at 7, 14, and 28 days are shown in Figure 1.

A total of 162 adverse events were recorded in 87 patients (65.4%), and the most frequent of which were sinus bradycardia (21, 13.1%) and alanine transaminase increase (18, 11.3%). All adverse events were classified as Grades 1–3, except one grade 4 hyperkalemia and one grade 4 acute kidney injury (Table 1).

## Discussion

The male predominance and the prevalence of chronic medical conditions in the study population in are consistent with Qatar's general demography and the previously described features of patients hospitalized with severe COVID-19.^10,11^

A high SARS-CoV-2 viral load is associated with increased COVID-19 severity.^12^. The clinical benefits from remdesivir are expected to reduce SARS-CoV-2 viral load in the respiratory tract. However, this phenomenon was not observed in a placebo-controlled randomized clinical trial of remdesivir for patients with severe COVID-19^13^ and an observational study from Israel.^14^

Critical COVID-19 is associated with poor clinical outcomes, especially among older individuals and those with multiple co-morbidities.^15^ In this single-arm study of adults with severe COVID-19 pneumonia requiring mechanical ventilation, remdesivir therapy was found to be associated with high clinical improvement rates and relatively low all-cause mortality by day 28. Several possible explanations can be offered for these findings. First, with its median age of 56 years, the study population is relatively younger than critically ill patients with COVID-19 reported elsewhere.^13,15,16^ Second, remdesivir therapy was started within a median of 7 days from onset of symptoms and 1 day from hospitalization. For maximized likelihood of benefit, antiviral therapy should be initiated as early as possible during the clinical course of COVID-19.^4-7^ The early initiation of remdesivir therapy in this cohort may have contributed to the observed positive outcomes. Third, all the patients received concomitant systemic corticosteroids, one of few interventions that can improve clinical outcomes and reduce mortality in critical patients with COVID-19.^2^ A recent population-based cohort study from Denmark showed a significantly reduced 30-day all-cause mortality associated with the combined use of remdesivir and dexamethasone in patients with COVID-19 requiring mechanical ventilation (weighted odds ratio 0.47, 95% CI 0.38–0.57).^17^ Meanwhile, the proportion of patients who received systemic steroids at the time of randomization was less than 40% in a negative randomized clinical trial of remdesivir for severe COVID-19 from China,^13^ and less than 50% of remdesivir recipients were included in the SOLIDARITY trial.^18^ In the present work, 24.8% of the patients received concomitant tocilizumab therapy, which may have contributed to the results. Unfortunately, the study sample size does not permit reliable subgroup analyses to determine the potential effect of adding tocilizumab to remdesivir and systemic corticosteroids.

The safety profile of remdesivir in this study is consistent with that in previous reports.^4^ One potential cause for concern is the relatively frequent, albeit non-severe, sinus bradycardia observed in the present work. Previous studies already reported the same finding,^19^ including some occasions of severe bradycardia.^20^ Awareness on this potential adverse event is important, especially for critically ill patients with COVID-19 who could have multiple potential causes for cardiac rate and rhythm abnormalities.

Our study is limited by its retrospective nature and the lack of a control arm. Admittedly, no firm conclusions should be drawn from single-arm descriptive studies. However, in the absence of high-tier clinical evidence, single-arm cohorts can provide some initial insights into the potential usefulness of a new agent for an emerging clinical entity.^21^ In particular, the present report provides some insights into the potential role of remdesivir in combination with systemic corticosteroids for patients with COVID-19 who require NIV or IMV, a group for whom data are severely limited. For example, ACTT-1 showed an overall association between remdesivir and short time to recovery, but this finding was not observed in the subset of patients who were on NIV (95 out of 541 participants, 17.6%) or IMV (131 out of 541 participants, 24%) at the time of treatment initiation; however, the study was not powered to address the primary outcome in these subgroups.^5^ Similarly, the research of Wang et al. was underpowered due to its premature discontinuation; the results are even less conclusive for the subset of patients who were on NIV (37 out of 236 participants, 15.7%) or IMV (1 out of 236 participants, 0.4%).^13^ Olender et al. reported a reduction in time to clinical recovery and all-cause mortality in a cohort of patients with severe COVID-19 who received remdesivir compared with a matched cohort who received standard care alone; however, only 19 out of 312 participants (6.1%) in the remdesivir arm and 20 out of 818 patients (2.4%) in the control arm were on mechanical ventilation.^22^ In the SOLIDARITY trial, only 254 out of 2,732 patients (9.3%) in the remdesivir arm and 233 out of 2,708 patients (8.6%) in the corresponding control arm were on mechanical ventilation. Notably, a breakdown by type of ventilation is not available in the present study.^18^ Although our report suggested the possible role of remdesivir in the clinical improvement of patients with severe COVID-19 pneumonia requiring high oxygen support, the findings cannot lead to any firm conclusions. Adequately powered randomized trials are warranted to establish whether remdesivir presents clinical benefits in patients with COVID-19 who require high levels of oxygen support.

## Conclusion

The use of remdesivir in combination with systemic corticosteroids is associated with high recovery rates and low all-cause mortality in patients with COVID-19 pneumonia who require NIV or IMV. The results need confirmation from randomized clinical trials of appropriate design and size.

### Authors’ Contribution

Conceptualization and study design: RSA, EZE, and ASO. Data curation: MZIE, ME, MA. Formal analysis and interpretation: RSA, EZE, JD, and ASO. Resources: MAA. Supervision: ASO. Writing–original draft preparation: RSA, EZE and ASO. All authors approved the version submitted for publication.

### Funding

No funding was required.

## Figures and Tables

**Table 1:** Adverse events associated with remdesivir use for patients with critical Coronavirus Disease 2019.

Adverse events	Count (%)

Total number of adverse events	162

Grade 4 and 5 adverse events	2 (1.2%)

Hyperkalemia	1 (50%)

Acute kidney injury	1 (50%)

Grade 1 to 3 adverse events	160 (98.8%)

Sinus bradycardia	21 (13.1%)

ALT increase	18 (11.3%)

Anemia	13 (8.1%)

Hypotension	12 (7.5%)

Diarrhea	10 (6.3%)

Hyperkalemia	10 (6.3%)

Hypophosphatemia	10 (6.3%)

AST increase	8 (5%)

abdominal pain	4 (2.5%)

Anorexia	4 (2.5%)

Hypokalemia	4 (2.5%)


ALT, alanine transaminase; AST aspartate transaminase
